# News Items of General Interest

**Published:** 1914-11

**Authors:** 


### News Items of General Interest.

---

Medical Society Entertains.—The Dallas Medical and Surgical Society entertained its members with a theater party and a buffet supper at the last meeting.

---

Dr. Morris H. Boerner, Assistant State Health Officer, and State Hookworm Commissioner, states that during the past quarter 6279 persons were examined and 19 per cent of this number were found to be infected with hookworm.

---

Annual Medical Convention to Be Held in Fort Worth May 3 to 5, 1915.—At the conference of the officers and committees of the State Medical Association, which was held in Fort Worth on October 22d, it was decided to hold the next annual meeting in Fort Worth next May.

---

The Bexar County Medical Society held a most interesting meeting on Thursday evening, October 22. Two interesting and instructive papers were read and discussed. One was by Dr. L. L. Shropshire, who spoke on "A New Vaginal Hysterectomy," and the other paper was by Dr. R. A. Goeth, who spoke on "Prevention and Treatment of Septicemia."

---

WOMAN SURGEON SELECTED.—Dr. Mary M. Crawford, of Brooklyn, has been chosen as one of six American surgeons selected for hospital and field service in France.

Dr. Crawford was born in Nyack, N. Y., in 1884. She was graduated from Cornell University in 1904.

---

MEDICAL ASSOCIATION MEETING.—The Hunt County Medical Association met in regular session October 9 in the county court room. The President, Dr. Waddle, presided. An excellent paper was read by Dr. M. L. Wilbanks, and also one by Dr. J. H. Gregory on "Cause and Prevention of Insanity."

---

THE Leon County Medical Society met October 7 at Normangee, and nearly every physician in the county was present. The following officers were elected for the ensuing year: Dr. S. R. Burroughs, of Buffalo, re-elected President; Dr. Joe Rogers, of Normangee, Vice-President; Dr. V. L. Smith, of Jewett, Secretary.

---

THE physicians of Fort Worth have devised the plan of having all candidates for municipal offices appear before them and deliver five-minute addresses concerning their ideas as to a board of health, city hospitals and general sanitary regulations. These addresses are taken down by a stenographer for future reference, lest the candidate forget his promises. This is a splendid idea and so practical that it can be used even in the smallest town.

---

MEDICAL EXAMINATIONS BY STATE BOARD.—Examinations for licenses to practice medicine in Texas will be held in Waco November 10, 11 and 12, by the State Board of Medical Examiners, of which Dr. W. L. Crosthwait, of Waco, is Secretary, and Dr. J. F. Bailey, President. The examinations will be held in the Y. M. B. L. rooms. Dr. Crosthwait expects about forty applicants to take the examination. The board will establish its headquarters at the Riggins Hotel.

ANNUAL MEETING OF THE NORTHWEST TEXAS MEDICAL ASSOCIATION.—The Northwest Texas Medical Association met in Jacksboro with about thirty members in attendance. The leading physicians from the district were in attendance. The day was spent in discussing scientific subjects.

Tuesday afternoon a number of automobiles were requisitioned and the delegation was taken out to the Jack county oil fields. At night the wives and daughters of the doctors and druggists of Jacksboro gave a banquet in the Woodman hall, and a most enjoyable occasion was held.

---

DOCTORS MEET.—The Milam County Medical Society met at the K. of P. hall in Rockdale Tuesday, October 13, 4 to 11 p. m. The following physicians were present: Drs. H. T. Coulter, S. B. Kirkpatrick, I. P. Sessions, T. S. Barkley and T. D. Roundtree, of Rockdale; J. C. Herring, of Burlington; D. E. Monroe, G. B. Taylor, I. M. F. Gill and E. E. Best, of Cameron; L. W. Kistland and G. W. Mullins, of Milano; D. F. Gray, of Gause; L. L. Lee and E. L. Lawrence, of Thorndale; J. C. Thomas, of Taylor; J. J. Terrell, Talley, Noble and McElroy, of Temple; L. H. Rodney, of Waco; C. S. Venable, of San Antonio; Burns, of Rodgers, who were entertained at the Hotel Wolf by local physicians, after which a good program was rendered.

---

INVISIBLE GERM IS DISCOVERED, SAYS DR. SIMON FLEXNER.—Dr. Simon Flexner, head of the Rockefeller Institute for Medical Research, who spoke at the Johns Hopkins Hospital anniversary celebration, voiced the belief that he has cultivated the hitherto invisible germ which causes infantile paralysis and has reproduced the disease in animals by injecting the culture of this germ.

Dr. Flexner has definitely succeeded in transmitting the disease to monkeys with the micro-organism which he had previously said he thought was the cause of the disease.

The doctor believes the disease is transmitted through the respiratory channels rather than by insects, as some have held, and he advanced the theory that healthy persons often carry the germ in their bodies and through them children are infected; that there are "carriers" of infantile paralysis just as there are "carriers" of typhoid and diphtheria who, while transmitting these diseases to others, do not suffer from them themselves.

Surgeon General Rupert Blue of the United States Public Health Service says: "There is no occasion for uneasiness about the closing of the baths abroad. In the first place, we have springs in this country that possess amazing curative properties, equal if not superior to those in Europe. People patronize those abroad because they are the fashion, not because of their superiority to the American cures. It's only another case of overlooking our own natural resources."

The fame of these springs is centuries old, but it was comparatively recent that their curative powers were found to depend upon the emanations of radium from the surrounding soil and mineral sub-strata.

Army and navy physicians in Washington, who are now handling radium in small quantities, supplement Dr. Blue's information with the statement that radium treatment in hospitals or under the direction of American physicians is really more effective than the spas, because of the fact that the individual dosage may be accurately measured.

The annual expenditures by American patients in European spas are well above $100,000,000. This will now be kept in the United States.

---

Army Medical Corps Examinations.—The Surgeon General of the Army announces that preliminary examinations for appointment of first lieutenants in the army medical corps will be held on January 11, 1915, at points to be hereafter designated.

Full information concerning these examinations can be procured upon application to the Surgeon General, U. S. Army, Washington, D. C. The essential requirements to secure an invitation are that the applicant shall be a citizen of the United States; shall be between 22 and 30 years of age, a graduate of a medical school legally authorized to confer the degree of Doctor of Medicine; shall be of good moral character and habits, and shall have had at least one year's hospital training as an interne, after graduation. The examinations will be held simultaneously throughout the country at points where boards can be convened. Due consideration will be given to localities from which applications are received, in order to lessen the traveling expenses of applicants as much as possible.

In order to perfect all necessary arrangements for the examinations, applications must be completed and in possession of the

Adjutant General at least three weeks before the date of examination. Early attention is, therefore, enjoined upon all intending applicants. There are at present twenty vacancies in the medical corps of the army.

---

THE Ellis County Medical Association convened in Midlothian Tuesday, October 5, at 2 p. m., Dr. Z. T. Bundy, of Milford, presiding.

The following physicians were present: Leonard Keplinger, Tom Cheatham, W. C. Tenery, G. W. Storm, S. H. Watson, L. D. Parnell, W. D. Boyd, all of Waxahachie; J. C. Loggins and W. P. McCall, of Ennis; Z. T. Bundy, of Milford; H. D. Nifong, of Britton; G. D. Weaver, of Ovilla; J. P. Harris, of Mountain Peak; P. L. Smith, of Houston; O. E. Moore, W. C. Brown and T. L. Barnett, of Midlothian.

Preceding the convening of the meeting the visiting doctors were guests of the local doctors at a lunch at the Mullen Hotel.

The first paper read was on "Tuberculosis," by Dr. L. Keplinger. Second, "Early Diagnosis and Treatment of Tuberculosis," by Dr. W. C. Brown. Third, "Diagnosis of Urgent Surgical Conditions of the Abdomen," by Dr. J. P. Harris. Discussion by Dr. W. C. Tennery.

Mrs. Zeb Rayburn, recently pronounced cured of pellagra, was presented, and a history of the case and its treatment presented by Dr. W. C. Brown. Mrs. Rayburn was examined and her case discussed and suggestions made in her case.

Dr. O. E. Moore presented Mrs. E. R. Scarbrough, another lady suffering with the same disease in an aggravated form, and gave history and treatment of case.

These cases and others were ably and interestingly discussed by Drs. Watson, McCall, Loggins and others.

Dr. T. L. Barnett reported a case of gall stones, complicated with malignant tumor of the liver, which was briefly discussed; after which the association adjourned to meet November 17 at Ennis.

---

THE NINTH ANNUAL MEETING of the Medical Association of the Southwest will be held in Galveston November 10 and 11. Dr. H. O. Sappington, Chairman of the Galveston Committee on Arrangements, says that from 300 to 400 doctors will attend the meeting. Preparations have been made by the local entertainment committee to give an oyster roast for the guests on Wednesday evening.

Below we give the program, hoping that every doctor in Texas who can possibly go will take advantage of this feast of good and helpful things.

Papers have been promised on the Section of General Medicine from Dr. L. J. Moorman, Oklahoma City, Okla.; Dr. G. Wilse Robinson, Kansas City, Mo.; Dr. Marvin C. Moore, Houston; Dr. John S. Turner, Dallas; Dr. A. R. Bowman, Uvalde; Dr. H. L. Wilder, Glen Ridge; Dr. D. C. Homan, Oglesby; Dr. J. M. Martin, Dallas; Dr. W. A. Hadley, Dickinson; Dr. J. H. Eastland, Mineral Wells; Dr. George H. Moody, San Antonio; Dr. John R. Worley, Dallas; Dr. Lee F. Watson, Oklahoma City, Okla.; Dr. F. Paschal, San Antonio; Drs. Singleton and Carter, Galveston; Dr. W. J. Calvert, Dallas; Dr. Ben E. Smith, Hillsboro; Dr. Julius McIver, Gainesville; Dr. Theo. Y. Hull, San Antonio; Dr. M. M. Smith, Dallas; Dr. J. O. Segura, Houston; Dr. West, Oklahoma City, Okla.; Dr. Riley, Oklahoma City; Dr. Carl Puckett, Pryor, Okla.

Papers on surgery have been promised by Dr. Gordon A. Beedle, Kansas City, Mo.; Dr. Albert Smith, Parsons, Kan.; Dr. Ernest G. Mark, Kansas City, Mo.; Dr. B. Belove, Kansas City, Mo.; Dr. W. D. McVicker, Wichita, Kan.; Dr. W. H. Stauffer, St. Louis, Mo.; Dr. F. C. Walsh, San Antonio; Dr. A. C. Scott, Temple; Dr. Arthur E. Sweatland, Nacogdoches; Dr. W. B. Russ, San Antonio; Dr. Jabez N. Jackson, Kansas City, Mo.; Dr. Walter S. Sutton, Kansas City, Mo.; Dr. R. C. Dorr, Batesville, Ark.; Dr. W. B. Thorning, Houston; Dr. J. Hutchings White, Muskogee, Okla.; Dr. Joe Thompson, Galveston; Dr. W. E. Dicken, Oklahoma City, Okla..; Dr. F. H. Clark, El Reno, Okla.; Dr. E. G. Blair, Kansas City, Mo.; Dr. A. B. Small, Dallas; Dr. G. Lester Hall, Kansas City, Mo.; Dr. K. A. Aynesworth, Waco; Dr. L. L. Shropshire, San Antonio.

Papers on the subject of the eye, ear, nose and throat have been promised by Dr. J. O. McReynolds, Dallas; Dr. E. H. Lanier, Texarkana; Dr. D. D. McHenry, Oklahoma City, Okla.; Dr. L. Haynes Buxton, Oklahoma City, Okla.; Dr. Flavel B. Tiffany, Kansas City, Mo.; Dr. W. G. Keibler, Goltry, Okla.; Dr. Edward H. Carey, Dallas; Dr. Horace R. Aynesworth, Waco; Dr. D. L. Bettison, Dallas; Dr. M. E. Taber, Dallas; Dr. W. E. Howard,

Dallas; Dr. J. G. Dorsey, Wichita, Kan.; Dr. Irvin Pope, Tyler; Dr. Harvey C. Haden, Galveston.

Special clinics will be arranged by Dr. Morris and Dr. Breath, of Galveston.

---

We wish to call the attention of those who are interested in sex problems to a new book which has just been issued by one of the JOURNAL'S contributing editors, Dr. Haverlock Ellis, of London, England.

For more than forty years Dr. Ellis has been devoting much time to this field of study, and to his book, "The Criminal," much credit is due for the protection that is today being thrown around the moral weakling, and the methods of reclamation in which the humane element is given a predominant place.

In 1898, Dr. Ellis published in England the "Psychology of Sex," which was immediately suppressed, although eminent physicians and men of science in many countries endorsed it as a scientific and reliable work.

Dr. Ellis secured a publisher in Germany and one in the United States and went steadily ahead with the investigations to which he had devoted his life, bringing out five volumes treating on various phases of this subject prior to 1907.

The sixth and concluding volume, "Sex in Relation to Society," has just been published in American by F. A. Davis & Co., of Philadelphia, and is in a sense a résumé of his forty years of study. We heartily recommend this book to all our readers.

